# Extracellular Matrix Stiffness and Composition Regulate the Myofibroblast Differentiation of Vaginal Fibroblasts

**DOI:** 10.3390/ijms21134762

**Published:** 2020-07-04

**Authors:** Alejandra M. Ruiz-Zapata, Andrea Heinz, Manon H. Kerkhof, Cindy van de Westerlo-van Rijt, Christian E. H. Schmelzer, Reinout Stoop, Kirsten B. Kluivers, Egbert Oosterwijk

**Affiliations:** 1 Department of Obstetrics and Gynecology, Radboud Institute for Molecular Life Sciences, Radboud University Medical Center, 6525 GA Nijmegen, The Netherlands; Cindy.vandeWesterlo-vanRijt@radboudumc.nl (C.v.d.W.-v.R.); Kirsten.Kluivers@radboudumc.nl (K.B.K.); 2 Department of Pharmacy, LEO Foundation Center for Cutaneous Drug Delivery, University of Copenhagen, 2100 Copenhagen, Denmark; andrea.heinz@sund.ku.dk; 3 Curilion, Women’s Health Centre, 2015 BJ Haarlem, The Netherlands; mhkerkhof@freeler.nl; 4 Department of Biological and Macromolecular Materials, Fraunhofer Institute for Microstructure Materials and Systems IMWS, 06120 Halle (Saale), Germany; schmelzer@pharmazie.uni-halle.de; 5 TNO Metabolic Health Research, 2301 DA Leiden, The Netherlands; reinout.stoop@tno.nl; 6 Department of Urology, Radboud Institute for Molecular Life Sciences, Radboud University Medical Center, 6525 GA Nijmegen, The Netherlands; Egbert.Oosterwijk@radboudumc.nl

**Keywords:** alpha-smooth muscle actin, cell culture system, collagen, decellularized tissues, extracellular matrix, matrix stiffness, micro-environment, myofibroblast, pelvic organ prolapse, vaginal fibroblasts

## Abstract

Fibroblast to myofibroblast differentiation is a key feature of wound-healing in soft tissues, including the vagina. Vaginal fibroblasts maintain the integrity of the vaginal wall tissues, essential to keep pelvic organs in place and avoid pelvic organ prolapse (POP). The micro-environment of vaginal tissues in POP patients is stiffer and has different extracellular matrix (ECM) composition than healthy vaginal tissues. In this study, we employed a series of matrices with known stiffnesses, as well as vaginal ECMs, in combination with vaginal fibroblasts from POP and healthy tissues to investigate how matrix stiffness and composition regulate myofibroblast differentiation in vaginal fibroblasts. Stiffness was positively correlated to production of α-smooth muscle actin (α-SMA). Vaginal ECMs induced myofibroblast differentiation as both α-SMA and collagen gene expressions were increased. This differentiation was more pronounced in cells seeded on POP-ECMs that were stiffer than those derived from healthy tissues and had higher collagen and elastin protein content. We showed that stiffness and ECM content regulate vaginal myofibroblast differentiation. We provide preliminary evidence that vaginal fibroblasts might recognize POP-ECMs as scar tissues that need to be remodeled. This is fundamentally important for tissue repair, and provides a rational basis for POP disease modelling and therapeutic innovations in vaginal reconstruction.

## 1. Introduction

Fibroblasts are key regulators of the repairing and remodeling of soft tissues throughout the body. When an injury occurs, fibroblasts migrate to the wound site and differentiate into myofibroblasts. Myofibroblasts are “transient” cells that are commonly identified using the markers: α-smooth muscle actin (α-SMA) present in their cytoskeleton, and by collagen I production [1]. Myofibroblasts help to close wounds in two ways: (i) by contracting the extracellular matrix (ECM), and (ii) by secreting large amounts of new ECM to fill in the gaps within the tissues [1,2]. The newly deposited immature scar, or granulation tissue, is stiffer than un-wounded tissues, and is characterized by increased amounts of collagen III relative to collagen I protein. The collagen I/III ratio is used as an indicator of the quality of the ECM [1]. 

Soft tissue stiffness and ECM composition can also be affected by disease. In the case of pelvic organ prolapse (POP; a female condition characterized by the weakening of the pelvic floor supportive tissues and subsequent protrusion of most commonly the bladder outside the body [3]), studies have found that the anterior vaginal wall tissues of these women are stiffer, compared to healthy tissues [4] and have affected fibroblasts that seem to be conditioned by the prolonged exposure to the abnormal prolapsed ECM [5,6,7,8,9,10]. The prolapsed vaginal micro-environment can further be affected when, during treatment for POP, vaginal reconstruction is performed using polypropylene meshes that are stiffer than the native tissues. Indeed, stiffer meshes used as treatment for POP have been shown to produce more adverse events [11,12,13], and it has recently been suggested in an animal model with POP, that construct stiffness is the main driver of treatment failure [14].

Matrix stiffness can promote myofibroblast differentiation in fibroblasts derived from several soft tissues, including skin and lungs [2,15], but whether or not, and to what extent, this is the case for vaginal fibroblasts is currently unknown. Another important feature in the micro-environment of the vaginal wall is the ECM protein content. The main ECM proteins of the vaginal wall are collagens I and III, elastin and glycosaminoglycans (GAGs) [16]. Several studies have tried to characterize the vaginal ECM protein content of prolapsed tissues reporting conflicting results, thus, the effect of POP on the ECM composition remains unclear [17,18,19,20,21,22,23,24,25].

Changes in the vaginal micro-environment can affect myofibroblast differentiation. Therefore, in this study, we wanted to investigate the effects of ECM stiffness and protein content on myofibroblast differentiation of vaginal fibroblasts derived from POP or healthy tissues. To this end, we employed a series of matrices with different stiffnesses in combination with vaginal fibroblasts from POP and control tissues to investigate if matrix stiffness regulates myofibroblast differentiation. To test the effect of ECM composition and specific stiffness, we performed biochemical and biomechanical characterizations of vaginal ECMs and created cell culture systems using decellularized vaginal tissues derived from women with and without POP. We show that, for vaginal fibroblasts, it is not only about stiffness, ECM composition is also required to regulate myofibroblast differentiation. We further provide preliminary evidence that the vaginal fibroblasts might recognize the ECM from prolapsed tissues as a scar tissues that need to be remodeled.

## 2. Results

### 2.1. Effect of Micro-Environments’ Stiffness on Vaginal Fibroblasts to Myofibroblast Differentiation

**Figure 1:**
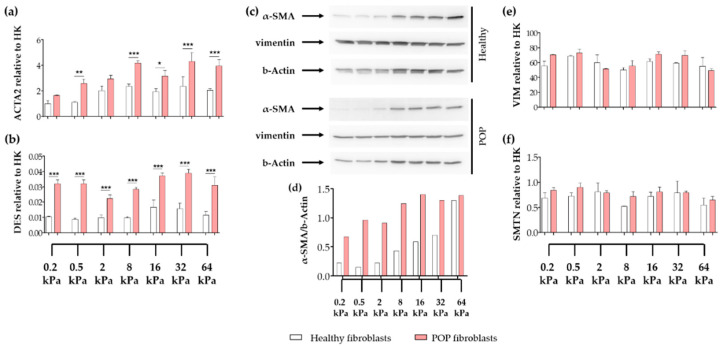
Expression of intracellular proteins of vaginal fibroblasts on different substrate stiffnesses. Healthy or pelvic organ prolapse (POP) fibroblasts were seeded for 48 h on collagen coated plates with specific stiffnesses: 0.2 kPa, 0.5 kPa, 2 kPa, 8 kPa, 16 kPa, 32 kPa or 64 kPa. The graphs show the relative gene expression to house-keeping genes (HK) of the genes: (**a**) *ACTA2* (alpha smooth muscle actin or α-SMA), (**b**) *DES* (desmin), (**e**) *VIM* (vimentin) and (**f**) *SMTN* (smoothelin). (**c**) Western blots showing protein levels of α-SMA, vimentin and b-Actin. (**d**) Corresponding quantification of the density of the bands of α-SMA normalized to b-Actin. Data represents the mean ± SD. * *p* < 0.05, ** *p* < 0.01, *** *p* < 0.001 by one-way analysis of variance (ANOVA), followed by Tukey’s multiple comparison test.

To test the hypothesis that vaginal fibroblasts will undergo myofibroblast differentiation as the micro-environment becomes stiffer, vaginal fibroblasts were seeded on surface micro-stiffnesses ranging from 0.2 kPa to 64 kPa, and the gene expression levels of the intracellular proteins α-SMA (gene *ACTA2*), desmin (gene *DES*), smoothelin (gene *SMTN*) and vimentin (gene *VIM*) were tested after 48 h of culture. Regardless of the substrate stiffness, the gene expression levels of *ACTA2* were higher in POP than in healthy fibroblasts (Figure 1a); whereas, *VIM* and *SMNT* showed no differences (Figure 1e,f). The gene expression levels of *ACTA2* steadily increased from 0.2 kPa to 8 kPa, where it reached a plateau. ACTA2 expression was positively correlated to stiffness in healthy (*r* = 0.4093; * *p* = 0.0327) and in POP fibroblasts (*r* = 0.5696; ** *p* = 0.0035). At the protein level (Figure 1c,d), α-SMA showed even a higher positive correlation to stiffness in healthy (*r* = 0.9862; *** *p* < 0.0001) and in POP fibroblasts (*r* = 0.6874; * *p* = 0.0440). In POP fibroblasts, this correlation was stronger from 0.2 kPa to 16 kPa (*r* = 0.9163; * *p* = 0.0143). The gene expression of *DES* was higher in POP than in healthy fibroblasts (Figure 1b), however, the expression levels were low, and we were unable to detect desmin by western blotting (data not shown). Gene and protein expressions of vimentin remained unchanged (Figure 1c,e), and were similar between control and POP cells.

**Figure 2:**
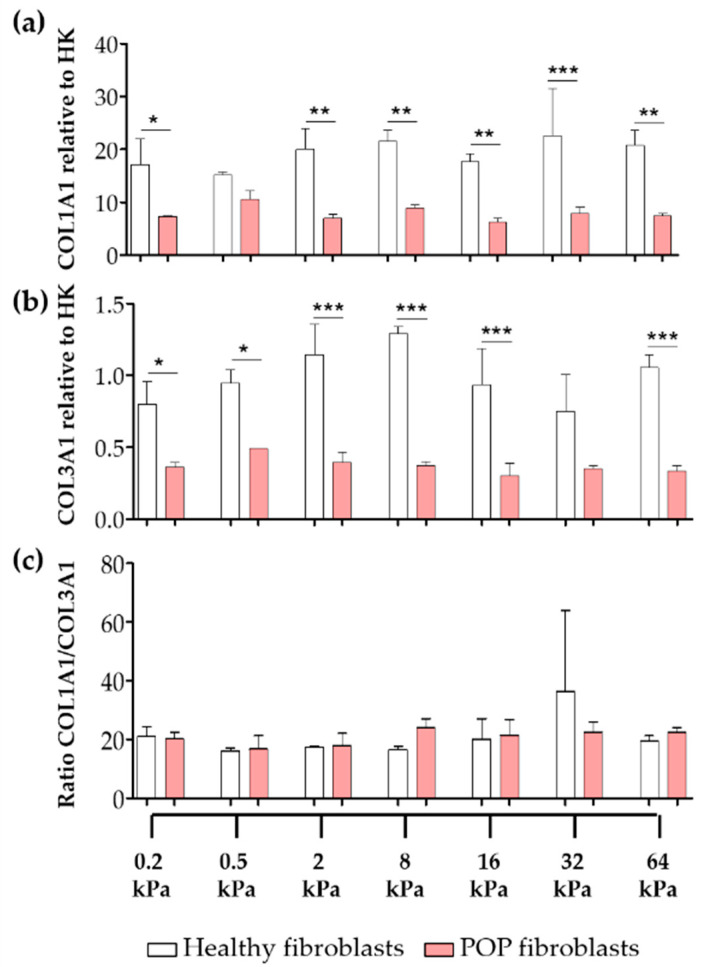
Gene expression of collagens of vaginal fibroblasts on different substrate stiffnesses. Healthy or POP fibroblasts were seeded for 48 h on collagen coated plates with specific stiffnesses: 0.2 kPa, 0.5 kPa, 2 kPa, 8 kPa, 16 kPa, 32 kPa and 64 kPa. The graphs show the relative gene expression to house-keeping genes (HK) of (**a**) *COL1A1* (collagen I) and (**b**) *COL3A1* (collagen III). (**c**) Ratio collagen I/III. Data represents the mean ± SD. * *p* < 0.05, ** *p* < 0.01, *** *p* < 0.001 by one-way ANOVA, followed by Tukey’s multiple comparison test.

Another important feature of fibroblast to myofibroblast differentiation is the excessive ECM deposition, mainly of collagens, by myofibroblasts into their surrounding micro-environment. The gene expression levels of *COL1A1* and *COL3A1* were up to three-times higher in healthy fibroblasts than in the POP fibroblasts (Figure 2). We found no correlation between the gene expression of the collagens and stiffness. There were no differences in between the ratios of collagen I/III gene expression (Figure 2c).

### 2.2. Biochemical and Biomechanical Characterization of Vaginal Extracellular Matrix (ECM)

Since stiffness alone was not enough to induce myofibroblast differentiation in vaginal fibroblasts, we looked at the composition of the vaginal ECM by performing biochemical and biomechanical characterizations of these natural scaffolds (Table 1). Regardless of the origin of the ECM, the collagen protein content was at least 17-times higher than the amount of elastin or the GAG proteins. The ECMs from tissues derived from POP patients had 30% more collagen and 91% more elastin protein content than ECMs derived from healthy tissues. Additionally, POP ECMs were 26% stiffer than healthy ECMs. There were no differences when comparing the ECM derived from POP and healthy tissues in the collagen cross-links pyridinolines and pentosidines, the elastin cross-link desmosine/isodesmosine, nor in the GAG content.

### 2.3. Vaginal Extracellular Matrix (ECM) Can Be Successfully Used as A Cell Culture System for Vaginal Fibroblasts

**Figure 3:**
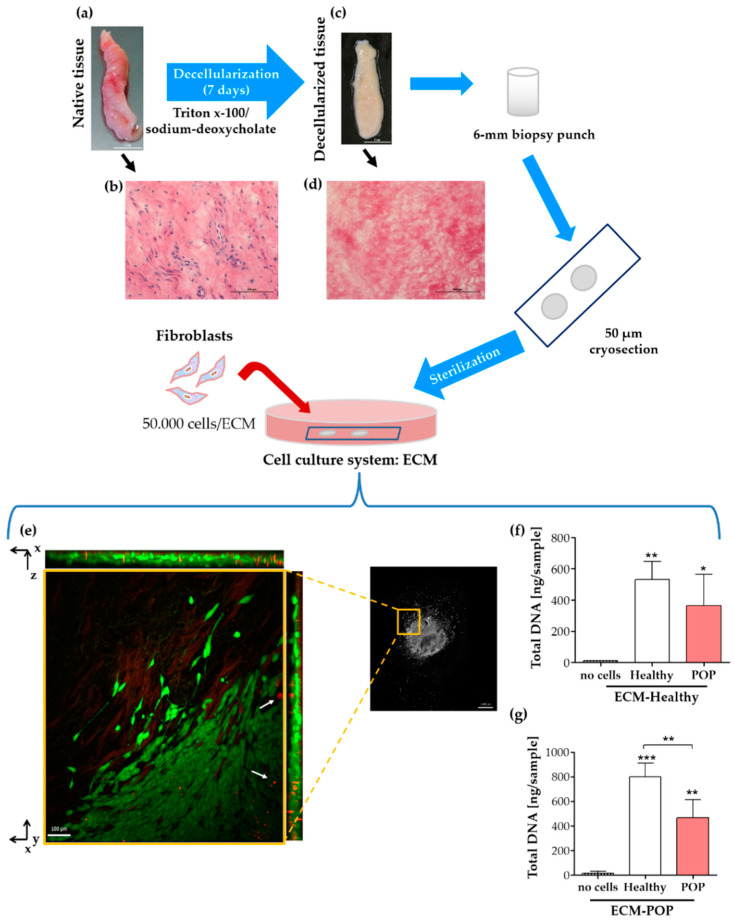
Cell culture system with vaginal extracellular matrix (ECM) and fibroblasts. The top figure shows a graphical representation of a cell culture system that was created using anterior vaginal biopsies (**a**,**b**). Native tissues were decellularized (**c**,**d**) and decellularization process was confirmed by haematoxylin-eosin staining of native (**b**) and decellularized tissues (**d**), where matrix is pink and the cell nuclei are blue. The white scale bars are 1 cm (**a**,**c**) and the black scale bars are 100 µm (**b**,**d**). Fibroblasts were seeded for 48 h on the ECMs, and viable cells were stained with the LIVE/DEAD^®^ Viability/Cytotoxicity kit (**e**), following manufacturer’s instructions (molecular probes, Life Technologies). (**e**) A close-up of the living fibroblasts (green) show that they can migrate into the ECMs in the z-plane (as shown in the orthogonal views) and also from the centre to the outside of the matrix. Dead cells (red, white arrows) were mainly found inside and towards the centre of the ECM. Images were acquired with a Zeiss Apotome.2 microscope with an apotome optical sectioner. The left image is an insert of the image on the right and is a maximum intensity projection with the orthogonal sections on the sides. The scale bar of the inserts is 100 μm and of the image on the right is 1 mm. Total DNA of healthy or POP cells seeded on ECM-Healthy (**f**) or ECM-POP (**g**) was measured. Data represents the mean ± SD. Differences between groups were calculated using One-way ANOVA, followed by Tukey’s multiple comparison test. * *p* < 0.05, ** *p* < 0.01 and *** *p* < 0.001 are compared to the first bar (no cells), unless otherwise indicated.

To test the effect of the natural vaginal scaffolds, ECM-Healthy and ECM-POP on vaginal fibroblasts’ behavior, we created a cell culture system using matrices from decellularized vaginal tissues (Figure 3). The efficacy of the decellularization process was macroscopically observed by the white aspect of the tissues compared to fresh biopsies (Figure 3a,c), and confirmed microscopically by the absence of nuclei within the decellularized tissues, as visualized by haematoxylin-eosin staining (Figure 3b,d). Seeded fibroblasts survived and migrated into the ECM as seen in the live/dead staining (Figure 3e). There was no difference in the amount of DNA between healthy and POP fibroblasts seeded on ECM-Healthy (Figure 3f). On the other hand, the number of healthy cells was higher than POP fibroblasts when seeded on ECM-POP, suggesting enhanced proliferation of healthy fibroblasts on ECM-POP (Figure 3g). The amount of DNA detected in the ECMs alone was lower than 15 ng/sample (Figure 3f,g; no cells).

### 2.4. Effect of Vaginal Extracellular Matrix (ECM) on Vaginal Fibroblasts’ Expression of Proteins Involved in Myofibroblast Differentiation

**Figure 4:**
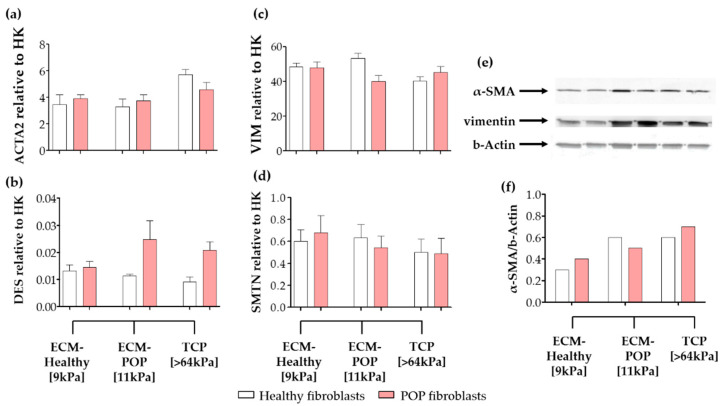
Expression of intracellular proteins of vaginal fibroblasts on different micro-environments. Healthy or POP fibroblasts were seeded for 48 h on different micro-environments: ECM-Healthy (vaginal decellularized extracellular matrix from healthy tissues), ECM-POP (vaginal decellularized extracellular matrix from pelvic organ prolapse tissues) or tissue culture plates (TCP). Relative gene expression to house-keeping genes (HK) of: (**a**) *ACTA2* (alpha smooth muscle actin or α-SMA), (**b**) *DES* (desmin), (**c**) *VIM* (vimentin) and (**d**) *SMTN* (smoothelin). (**e**) Western blots showing protein levels of α-SMA, vimentin and b-actin. (**f**) Corresponding quantification of the density of the bands of α-SMA normalized to b-actin. Data represents the mean ± SD. * *p* < 0.05, ** *p* < 0.01, *** *p* < 0.001 by one-way ANOVA, followed by Tukey’s multiple comparison test.

There were no significant differences in the gene expression levels of the intracellular proteins *ACTA2*, *DES*, *VIM* and *SMTN* between healthy and POP fibroblasts seeded on vaginal ECMs (Figure 4a–d). Interestingly, the gene expression levels of *ACTA2* (Figure 4a), for both healthy and POP fibroblasts, were within the range of the expression detected, when POP fibroblasts were exposed to similar surface micro-stiffnesses (8 kPa to 16 kPa; Figure 1a). For healthy fibroblasts, *ACTA2* gene expression levels were higher when cells were seeded on ECMs (Figure 1a), compared to the same cells seeded on equivalent stiffnesses (8 kPa to 16 kPa; Figure 1a): 6-fold in ECM-Healthy and 8-fold in ECM-POP. At the protein level, α-SMA in control fibroblasts was higher when the cells were cultured on ECM-POP (stiffness of 11 kPa; Figure 4e,f) and in tissue culture plates (TCP; stiffness > 64 kPa) than when they were cultured on ECM-Healthy (stiffness of 9 kPa; Figure 4e,f). In POP fibroblasts, α-SMA was similar in all conditions (Figure 4f). The gene expression levels of *DES* were again low, and we were unable to detect desmin by western blotting (data not shown). 

**Figure 5:**
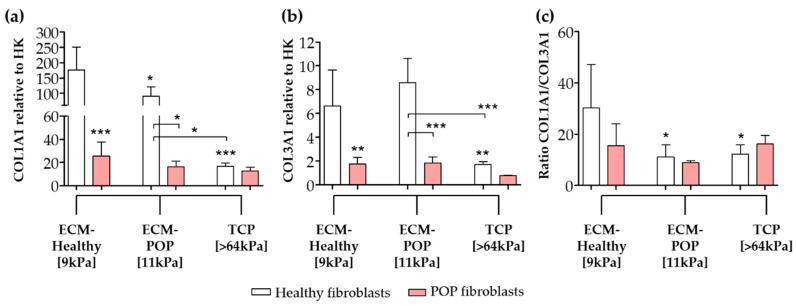
Gene expression of collagens of vaginal fibroblasts on different micro-environments. Healthy or POP fibroblasts were seeded for 48 h on different micro-environments: ECM-Healthy (vaginal decellularized extracellular matrix from healthy tissues), ECM-POP (vaginal decellularized extracellular matrix from pelvic organ prolapse tissues) or TCP (tissue culture plates). The graphs show the relative gene expression to house-keeping genes (HK) of collagen I ((**a**); *COL1A1*) and collagen III ((**b**); *COL3A1*). (**c**) Ratio of *collagen I/III*. Data represents the mean ± SD. * *p* < 0.05, ** *p* < 0.01, *** *p* < 0.001 by one-way ANOVA, followed by Tukey’s multiple comparison test.

The gene expression levels of the extracellular proteins *COL1A1* and *COL3A1* were higher in healthy fibroblasts seeded on ECMs and compared to TCP or to POP cells (Figure 5). *COL1A1* was higher when cells were seeded on ECMs (Figure 5a), compared to the same cells seeded on matrices with equivalent stiffness (8 to 16 kPa; Figure 2a). Healthy fibroblasts seeded on ECM-Healthy showed the highest *COL1A1* gene expression (Figure 5a). Healthy fibroblasts seeded on ECM-Healthy and ECM-POP showed similar levels of *COL3A1* gene expression, which were higher than the same cells seeded on TCP (Figure 5b). The ratio of collagen I/III gene expression was lower when healthy fibroblasts were seeded on ECM-POP, compared to ECM-Healthy (Figure 5c). *COL3A1* gene expression levels were higher when cells were seeded on ECMs (Figure 5b), compared to the same cells seeded on matrices of equivalent stiffness (8 kPa to 16 kPa; Figure 2b). The gene expression levels of collagens I and III of POP fibroblasts seeded on vaginal ECMs were not different to the same cells seeded in TCP (Figure 5a,b).

## 3. Discussion

Understanding the effect of stiffness and matrix composition on vaginal fibroblast to myofibroblast differentiation is important to further our knowledge about vaginal tissue repair, shed light into the development of pelvic floor disorders and help improve future biomaterial design for vaginal reconstruction. 

Fibroblast to myofibroblast differentiation plays an important role in normal and pathological soft tissue repair and remodeling [26]. This process can be triggered by increasing the stiffness in the fibroblast’s micro-environment of soft tissues, including the skin and lungs [2,15]. Here, we studied the effect of stiffness on myofibroblast differentiation of vaginal fibroblasts. We showed that stiffness alone was not enough to induce myofibroblast differentiation of vaginal fibroblasts, since increased stiffness induced the production of alpha-smooth muscle actin (α-SMA), but not of collagens. We also showed that vaginal extracellular matrix (ECMs) induced myofibroblast differentiation, since both α-SMA and collagen I production were increased. Moreover, collagen I/III gene expression ratio was lower when fibroblasts were seeded on vaginal ECMs from prolapsed tissues. 

A possible reason for the observed phenotypical changes in vaginal fibroblasts is that the cells identify the changes in the prolapsed ECMs as resembling those from scar or granulation tissues. Granulation tissues are laid down by myofibroblasts as part of the normal wound-healing process [1]. These tissues are stiffer [27], and have a lower collagen I/III ratio, compared to un-wounded tissues [28]. The ECMs derived from women with pelvic organ prolapse (POP) that were used in this study seem to have the characteristics of scar tissues, since they were stiffer than healthy controls and had higher ECM protein content (collagen and elastin). Others have also reported increased stiffness of POP tissues [4], and some have reported a lower collagen I/III ratio [19,29,30]. Here, we provide evidence that, in the absence of other cells, vaginal fibroblasts recognize the stiff, collagen rich prolapsed ECMs as granulation tissues that need to be remodeled and, as a consequence, myofibroblast differentiation is triggered. Indeed, when vaginal fibroblasts were exposed to the altered prolapsed vaginal ECMs, they differentiated into myofibroblasts with a lower collagen I/III gene expression ratio, than when cells were seeded on healthy ECMs. The altered tissue micro-environment from women with POP may contribute to the maintenance of POP by regulating vaginal fibroblast to myofibroblast differentiation. A prolonged exposure of vaginal fibroblasts to an abnormal prolapsed matrix seems to skew the cells towards a different phenotype [5,6,7,8,9,10]. There is more and more evidence suggesting that vaginal fibroblasts from women with POP have phenotypical changes that seem to be conditioned by a prolonged exposure to the abnormal prolapsed matrix [5,6,7,8,9]. Since they respond differently than healthy cells, they might not be able to restore the ECM in the correct manner, thus creating a positive feed-back loop, where the matrix and cells influence each other in a vicious cycle. This could lead to an additional loss of strength, increased stiffness and, eventually, more tissue damage, contributing to the perpetuation of POP [6,7].

When and how the tissues become abnormal, and cells make the switch from a normal vaginal fibroblast to an affected POP phenotype, remains unclear. Here, we show specifically that the production of α-SMA in control fibroblasts is triggered by changes in both the ECM content and the surface micro-stiffness; whereas in POP fibroblasts, surface micro-stiffness seems to be the main driver of production of α-SMA. This higher mechano-sensitivity to surface stiffness by POP fibroblasts has recently been observed by others [31]. 

What could be an explanation for this phenomenon? 

Fibroblasts derived from POP tissues seem to be sensitized towards a synthetic phenotype characterized by an even higher α-SMA gene expression, compared to healthy cells seeded on surroundings with the same micro-stiffness. The *COL1A1* and *COL3A1* gene expressions in POP fibroblasts were lower than in healthy cells, regardless of the micro-environment. Cells with increased α-SMA content but that are low in collagen I expression are characteristic of a contractile phenotype of smooth muscle cells, and can be differentiated from myofibroblasts based on high smoothelin expression [32]. The cells studied expressed similar and relatively low smoothelin levels, regardless of whether or not the cells came from diseased tissues, or if they were on different surface micro-stiffness. Therefore, these cells do not exhibit the contractile phenotype characteristic of smooth muscle cells. Our observations are in line with previous studies, which showed that fibroblasts from women with POP have lower contractile capacities than healthy controls [5,6]. This provides additional evidence that vaginal fibroblasts from prolapsed tissues do not have a contractile phenotype. A better characterization of the fibroblasts’ phenotypical changes that occur in prolapsed tissues from women with POP would shed light on this enigma.

Fibroblasts’ behavior depends on the surrounding matrix, and POP and healthy cells behave in different ways when exposed to prolapsed matrices, the effect of matrix components and growth factors is worth exploring in a disease-specific manner. It would also be of great relevance to identify which matrix components dictate the phenotype of the fibroblasts and the dynamics of this process under normal and pathological conditions. Future studies could use variations of our complex in vitro models to investigate the effect of specific changes in ECM proteins (e.g., elastin, collagen III, fibrin, GAGs, etc), in combination with different stiffnesses and specific factors that are known to regulate cellular behavior in the vaginal micro-environment and that have been related to POP (e.g., oestrogens, transforming growth factor-β1, platelet rich plasma, etc.). Understanding cell-matrix interactions in the vaginal tissue not only would help us unravel the molecular mechanisms of POP, but could also be used to test novel treatments, such as tissue engineering. 

From the perspective of vaginal tissue engineering, the biochemical and biomechanical properties of the biomaterials should be taken into account. Currently, vaginal reconstruction of the anterior wall may involve using stiff transvaginal meshes as a treatment for POP. This treatment remains controversial, and on 16 April 2019, the food and drug administration (FDA) ordered all manufacturers to stop selling and distributing their products in the United States of America immediately [12]. To date, vaginal meshes have been banned in Australia, New Zealand and the United Kingdom [33]. There is a need for new materials that not only restore the anatomical function, but also optimize the local environment of the pelvic floor, to re-establish the proper functioning of the supportive tissues and the cells involved. As we have shown in the case of the vaginal micro-environment, it is not only about stiffness. The ECM protein content influences vaginal fibroblast behavior and, in particular, regulates myofibroblasts differentiation. Since fibroblast to myofibroblast differentiation plays a key role in tissue remodeling, and good graft-tissue integration depends on it, understanding how ECM stiffness, protein content and other stimuli regulate vaginal fibroblast behavior will help us to improve the design of vaginal graft biomaterials. 

Other soft tissue diseases from the genitourinary tract, such as mucosal atrophy, endometriosis or cancer, could also be studied using variations of our models. These complex in vitro models could be adapted by using cells derived from patients with those diseases, and could be used as screening tools for possible treatments. These screenings can be designed to be personalized by using cells and matrices from the patient requiring the treatment. 

## 4. Materials and Methods

### 4.1. Biopsy Collection, Cell Isolation and Tissue Decellularization

Full thickness biopsies (>1 cm^2^) from the anterior vaginal wall were retrieved from women with POP or healthy controls who were operated on for benign gynaecological indications. Biopsies were collected in PBS at 4 °C, and within 24 h, cells were isolated, or tissues were decellularized. Primary human vaginal fibroblasts were isolated, as previously described [7]. Experiments were performed with cells from passages 4–6, cultured in Dulbecco’s modified Eagle’s medium (DMEM; Gibco, Thermo Fisher, San Jose, CA, USA) supplemented with 1 g/L glucose, 10% foetal calf serum (FBS; HyClone, South Logan, UT, USA), 1% penicillin/streptomycin (100 U/mL penicillin and 100 µg/mL streptomycin; Sigma-Aldrich, St. Louis, MO, USA) and 1% fungizone (Gibco, Thermo Fisher, San Jose, CA, USA). Tissues were decellularized using a Triton x-100/sodium-deoxycholate treatment [34]. Biopsies were incubated at 37 °C and constantly agitated for 24 h in 0.25% w/w Triton x-100 and 0.25% w/w sodium-deoxycholate (Sigma-Aldrich, St. Louis, MO, USA) in PBS, washed with DMEM (Gibco, Thermo Fisher, San Jose, CA, USA) for 72 h at 4 °C and treated with DNase I (150 IU/mL) with 50 mmol MgCl_2_ (Sigma-Aldrich, St. Louis, MO, USA) in PBS for 24 h at 37 °C. Samples were then incubated in DMEM for 24 h at 4 °C, extensively washed with PBS and incubated for a further 24 h at 37 °C in 1% penicillin/streptomycin PBS (100 U/mL penicillin and 100 µg/mL streptomycin; Sigma-Aldrich, St. Louis, MO, USA). Decellularized biopsies were kept in PBS at 4 °C, until further use. Tissue collection was approved by the medical ethical committee of the VU University Medical Centre (Amsterdam; IRB approval by METc VUmc registration number 2007/153, date 01/11/2007), and informed consent was obtained from all participants. 

### 4.2. Matrices with Different Stiffness for Cell Culture 

As matrices with different surface micro-stiffnesses, we used 6-well CytoSoft^®^ plates with different micro-stiffnesses (0.2 kPa, 0.5 kPa, 0.8 kPa, 2 kPa, 8 kPa, 16 kPa, 32 kPa and 64 kPa) were coated with PureCol^®^ Type I collagen, following manufacturer’s instructions (Advanced Biomatrix, San Diego, CA, USA). Cells were seeded at a cell density of 1 × 10^5^ cells/well for 48 h. Samples were collected for quantitative real time-polymerase chain reaction (RT-PCR) or for western blotting analysis (see below).

### 4.3. Haematoxylin and Eosin Staining (H&E)

Haematoxylin and Eosin (H&E) staining was performed on vaginal tissues before and after decellularization. Samples were snap frozen in Tissue-Tek (Sakura Finetek, Zoeterwoude, The Netherlands) kept in liquid N_2_, cryosectioned in series of 10 µm on the sagittal plane, mounted on poly-lysine coated slides and fixed for 10 min in 4% formaldehyde, prior to H&E staining. Images were acquired with a Leica DMRA microscope, equipped with a DFC300FX camera and using QWinPro software (Leica Microsystems, Switzerland).

### 4.4. Biochemical Analyses of Collagen

Tissues were lyophilized, weighed and hydrolyzed in 6 M HCl at 95 °C for 20 h. The amount of collagen was determined using the Quickenzyme total collagen assay (Quickzyme Biosciences, The Netherlands). The mature collagen cross-links (pyridinolines and pentosidines) were measured as previously described [35], using a reverse-phase high performance liquid chromatography (HPLC) system, equipped with online sample purification on CC31 cellulose, using a Prospekt solid-phase extractor (Separations, Jasco Benelux BV, The Netherlands) and expressed as crosslinks per triple helix. 

### 4.5. Biochemical Analyses of Elastin

Tissues were washed with PBS and stored at 5 °C in milliQ water with sodium azide (Sigma-Aldrich, St. Louis, MO, USA) until elastin isolation was performed. Samples lyophilized (SpeedVac, Thermo Fisher, St. Jose, CA, USA) and weighed before elastin isolation, which was performed as described earlier [36]. To summarize, tissue samples were subjected to treatment with a variety of reagents including organic solvents, cyanogen bromide, formic acid, mercaptoethanol and urea (Sigma-Aldrich, St. Louis, MO, USA), to remove all components present in the tissue except for elastin, which is resistant to this procedure. For the proteolysis of elastin, the elastin samples were dispersed in 50 mM Tris buffer (Merck, Darmstadt, Germany), pH 7.5 at a concentration of 1 mg/mL. Samples were digested with pancreatic elastase (Elastin Products Company, Owensville, MO, USA) for 24 h at 37 °C, using enzyme-to-substrate ratios of 1:100 (*w*/*w*). All digestions were stopped by adding trifluoroacetic acid (Sigma-Aldrich, St. Louis, MO, USA) to a final concentration of 0.5% (*v*/*v*). The analysis of the enzymatic digests of elastin samples was carried out according to a protocol published before [37]. NanoHPLC-nanoESI-QqTOF MS/MS was performed using an UltiMate 3000 nanoHPLC system (Thermo Fisher, San Jose, CA, USA), coupled to a QqTOF mass spectrometer Q-TOF-2 (Waters/Micromass, Manchester, UK). The chromatographic separation of the peptides was performed at 40 °C, using a flow rate of 300 nl/min. The operating conditions for the mass spectrometer during MS/MS measurements were chosen, as described earlier [35] except for the sample cone voltage, which was lowered to 20 V to minimize in-source decay. For full scan MS measurements, the samples were analysed in random order, and each sample was measured seven times. Data was acquired in the m/z range between 40 and 1550. Peptide sequencing was performed by automated de novo sequencing of the nanoESI-QqTOF MS/MS data, followed by database matching using the software Peaks Studio (version 7; Bioinformatics Solutions, Waterloo, Canada) [38]. The searches were taxonomically restricted to *Homo sapiens* and the enzyme was set to ‘none’. The formation of hydroxylated proline residues was considered a variable modification. Mass tolerances for precursor and fragment ions were set to 40 ppm and 0.1 Da, respectively. For statistical analysis of MS data using the Progenesis QI software (Non-linear Dynamics, UK), nanoESI QqTOF MS/MS data was imported into the software and converted into a single mgf. The mgf was submitted to a local Mascot server (Matrix Science, UK), and peptides were sequenced using the settings described above. The quantification of the tetrafunctional elastin cross-links desmosine and isodesmosine was performed using a LC-MS analysis using an Agilent 1100 LC system (Agilent, Germany), coupled to a quadrupole ion trap mass spectrometer Finnigan LCQ (Thermo Fisher, San Jose, CA, USA) via an electrospray interface, using the settings previously described [37]. 

### 4.6. Biochemical Analyses of Glycosaminoglycans (GAG) 

The amount of GAG was measured on tissues that were freeze-dried, weighed and hydrolyzed with 6 mol/L hydrogen chloride at 110 °C for 20 h, following a previous protocol [39]. The standard curve was prepared from chondroitin sulphate from shark cartilage (Sigma, St. Louis, MO, USA). 

### 4.7. ECM for Cell Culture: Preparation and Reseeding 

Decellularized vaginal tissues were used as ECM for cell culture. To keep the culture system as homogeneous as possible, we made cylinders on the transversal plane of the decellularized vaginal tissues with a sterile 6-mm biopsy punch. Biopsies were snap-frozen in Tissue-Tek (Sakura Finetek, Zoeterwoude, The Netherlands), cut in series of 50 µm thick and mounted on poly-lysine coated slides (two samples per slide). Sections were dried for 1 h at room temperature and washed with PBS, then placed in a petri dish, sterilized by a quick wash with 70% ethanol solution, and kept at 4 °C in 1% penicillin/streptomycin PBS until further use. Fibroblasts were counted, re-suspended in supplemented DMEM medium at a concentration of 1 × 10^7^ cells/mL, seeded on top of the ECM (5 µL/matrix, 1 × 10^7^ cells/mL) and incubated for 30 min at 37 °C. Supplemented DMEM medium was then carefully added to the 10 cm petri dishes. Cells were cultured for 48 h and viable cells were stained with the LIVE/DEAD^®^ Viability/Cytotoxicity kit, following the manufacturer’s instructions (molecular probes, Life Technologies, Paisley, UK). Viable cells were imaged with a Zeiss Apotome.2 microscope (Carl Zeiss, Oberkochen, Germany). Extra samples were also collected for total DNA, quantitative RT-PCR, or western blot analysis (see below). Cells seeded on regular tissue culture 24-well plates were used as positive controls, and unseeded ECMs were used as negative controls. 

### 4.8. ECM Surface Micro-Stiffness 

The mechanical properties of the ECMs were measured using a micro-indentor designed to test the surface micro-stiffness of biological tissues (PIUMA, Optics11, The Netherlands). Samples were indented in PBS with the PIUMA device, using a soft cantilever on the top of a ferruled optical fiber, with a radius of 95 µm and a stiffness of 0.5 N/m. The indentations were depth controlled (15 µm) and the loading and unloading periods were set to 3 s. Based on the load and displacement curves, the effective Young’s modulus was automatically calculated by the software from the PIUMA (Optics11, v1). A stiff surface was used before each series of tests for calibration. 

### 4.9. Total DNA 

Samples were homogenized in 350 µL of Lysis-M buffer, including proteinase inhibitor (Roche Diagnostics GmbH, Mannheim, Germany). After three freeze and thaw cycles, total DNA was measured with the commercial CyQuant cell proliferation assay kit, according to the manufacturer’s instructions (Molecular Probes Inc., Life Technologies, Paisley, UK). Fluorescence was measured using the Synergy^TM^HT multi-mode microplate reader (Biotek Instruments Inc., Vermont, USA). 

### 4.10. Quantitative RT-PCR 

Total RNA was isolated using TRIzol, and following the manufacturer’s instructions (Invitrogen, Life Technologies, Auckland, New Zealand). Concentration and purity of the RNA were determined with a Nanodrop-1000 spectophotometer (Fisher Scientific, Waltham, MA, USA). Then, 2 μg DNase-I-treated total RNA was used to make cDNA using random hexamer primers (Roche, Mannheim, Germany) and SuperScript II Reverse Transcriptase (Invitrogen, Life Technologies, Paisley, UK). RT-PCR was performed with a LightCycler 480 SYBR Green I Master mix and in a Light Cycler LC480 device (Roche, Mannheim, Germany), with the following amplification conditions: 5 min at 95 °C, followed by 45 cycles of 10 s at 95 °C, 20 s at 60 °C, 20 s at 72 °C. Crossing-point (Cp) values were determined using the LightCycler480 SW 1.5 software (Roche, Mannheim, Germany). Relative gene expression levels were normalized to two house-keeping genes (*YWHAZ* and *HPRT*), and were calculated according to the mathematical model for relative quantification in RT-PCR described by Pfaffl [40]. Primers were designed using Primer-BLAST, are listed in Table 2 and were acquired from Life technologies (Paisley, UK). Only primers on intended targets and separated by at least one intron were selected, and the integrity of the PCR products was confirmed by Sanger sequencing. 

### 4.11. Western Blot Analysis 

Proteins were extracted directly from cells cultured in the Cytosoft 6-well plates with Laemmli buffer containing β-mercaptoethanol and denatured for 5 min at 100 °C. The decellularized tissues were transferred to Eppendorf tubes before extracting and denaturing the proteins. The samples were fractionated by SDS-PAGE using 13% acrylamide, and transferred onto PVDF membranes. Blots were blocked for 2 h at room temperature with a blocking buffer (5% milk in PBS with 0.1% Tween-20 (PBST); note: for desmin, no Tween-20 was added). They were then incubated with the primary antibodies anti-α-SMA mouse monoclonal antibody (clone 1A4, Sigma, St. Louis, MO, USA; #A-2547, 1:5000), anti-vimentin mouse monoclonal antibody (RV202, BD Pharmingen, BD Biosciences, San Jose, CA; #550513, 1:5000) and anti-β-actin mouse monoclonal antibody (AC-15, Sigma, St. Louis, MO, USA; #A5441, 1:5000) diluted in 0.5% milk in PBST or the primary antibody anti-desmin mouse monoclonal antibody (clone D33, DAKO, Copenhagen, Denmark; #M0760, 1:100) diluted in 10% goat serum in PBST for 2 h at room temperature. After washing with PBST, blots were either incubated for 1 h at room temperature with a secondary sheep anti-mouse IgG antibody (1:50,000; Amersham ECL mouse IgG, HRP-linked whole Ab from sheep, Invitrogen, Life Technologies, Paisley, UK; #NA931), or for 30 min with a secondary goat anti-mouse IgG antibody (1:5000; goat anti-mouse IgG (H+L) secondary antibody, DyLight800, Thermo scientific, Rockford, IL, USA; #SA5-35521). After washing with PBST, bound antibodies were visualized either with the Amersham ECL prime western blotting detection reagent (Invitrogen, Life Technologies, Paisley, UK;#RPN2232) on the ImageQuant LAS4000 imaging system, or at 800 nm using the Odyssey CLx imaging system (LI-COR Biosciences, Nebraska, USA). Quantification of band densities was performed using ImageJ1.44p software (NIH). The original western blot images for Figure 1 can be found in Supplementary Figure S1 and for Figure 4 in Supplementary Figure S2.

### 4.12. Statistical Analysis 

Data are expressed as mean ± standard deviation (SD). Statistical analysis was performed using two-tailed unpaired *t*-test test, or one-way analysis of variance (ANOVA), followed by Tukey-Kramer’s post-hoc test (Prism version 5.02, GraphPad Software Inc., La Jolla, CA, USA). Correlation coefficients between parameters were evaluated by Pearson correlation using Prism (version 5.02, GraphPad Software Inc., La Jolla, CA, USA). Differences were considered significant at 5% level (*p* < 0.05). 

## 5. Conclusions

Our findings shed light on how matrix stiffness and protein content regulate myofibroblast differentiation in vaginal fibroblasts. We specifically show that vaginal fibroblast differentiation to myofibroblasts is not only directed by ECM stiffness, but that changes in the ECM composition are key regulators. We further provide preliminary evidence that the vaginal fibroblasts might recognize the ECM from prolapsed tissues as a scar tissues that need to be remodeled. These findings are fundamentally important for tissue repair and regeneration, and provide a basis for POP disease modelling and therapeutic development of vaginal reconstruction.

## Figures and Tables

**Table 1:** Biomechanical and biochemical characterization of vaginal extracellular matrix (ECM).

Feature	ECM-Healthy	ECM-POP	*p* Value
Collagen ^a^	84.3 (± 15.63)	110.0 (± 8.69)	0.0099 **
Pyridinolines per collagen molecule (HP + LP)/triple helix	0.11 (± 0.03)	0.14 (± 0.02)	0.0525
Pentosidine/triple helix	0.002 (± 0.0009)	0.001 (± 0.0006)	0.0606
GAG ^b^	3.7 (± 0.43)	3.6 (± 1.05)	0.8471
Elastin ^c^	3.4 (± 0.67)	6.5 (± 1.37)	0.0020 **
Desmosine/Isodesmosine	17.6 (± 7.54)	20.7 (± 2.14)	0.4068
Effective Young modulus (kPa)	8.9 (± 4.26)	11.2 (± 5.37)	0.0123 *

ECM: extracellular matrix; POP: pelvic organ prolapse; HP: hydroxylysinepyridinoline; LP: lysylpyridinoline; GAG: glycosaminoglycans. Data are in µg/mg of wet weight (a), µg/mg of dry weight (b), or in µg/mg of elastin (c), and are presented as: mean (± SD). *p*-values were calculated with two-tailed unpaired *t*-test. * *p* < 0.05, ** *p* < 0.01.

**Table 2:** Primer sequences used for real time-polymerase chain reaction (RT-PCR).

Target Gene		Oligonucleotide Sequence
*ACTA2*	Forward Reverse	5′ CCTGACTGAGCGTGGCTATT 3′ 5′ GATGAAGGATGGCTGGAACA 3′
*COL1A1*	Forward Reverse	5′ TCCAACGAGATCGAGATCC 3′ 5′ AAGCCGAATTCCTGGTCT 3′
*COL3A1*	Forward Reverse	5′ GATCCGTTCTCTGCGATGAC 3′ 5′ AGTTCTGAGGACCAGTAGGG 3′
*DES*	Forward Reverse	5′ TGCATGAAGAGGAGATCCGTG 3′ 5′ GGCAGTGAGGTCTGGCTTAG 3′
*HPRT*	Forward Reverse	5′ GCTGACCTGCTGGATTACAT 3′ 5′ CTTGCGACCTTGACCATCT 3′
*SMTN*	Forward Reverse	5′ CTATGGGCAGCTTAGCCCTC 3′ 5′ CATGGGTCTCCGCAGATGAG 3′
*VIM*	Forward Reverse	5′ GGCTCAGATTCAGGAACAGC 3′ 5′ GCTTCAACGGCAAAGTTCTC 3′
*YWHAZ*	Forward Reverse	5′ GATGAAGCCATTGCTGAACTTG 3′ 5′ CTATTTGTGGGACAGCATGGA 3′

*ACTA2*, alpha-smooth muscle actin; *COL1A1*, α1(I)procollagen; *COL3A1*, α1(III)procollagen; *DES*, desmin; *HPRT*, hypoxanthine phosphoribosyltransferase; *SMTN*, smoothelin; *VIM*, vimentin; *YWHAZ*, tyrosine 3-monooxygenase/tryptophan 5-monooxygenase activation protein, zeta polypeptide.

## Supplementary Materials

The following are available online at https://www.mdpi.com/1422-0067/21/13/4762/s1.

## Abbreviations

ANOVA	One-Way Analysis of Variance
α-SMA	α-Smooth Muscle Actin
Cp	Crossing-point
DMEM	Dulbecco’s Modified Eagle’s Medium
DNA	Deoxyribonucleic Acid
ECM	Extra Cellular Matrix
FCS	Foetal Calf Serum
FDA	Food and Drug Administration
GAG	Glycosaminoglycans
HK	House-Keeping
HP	Hydroxylysinepyridinoline
HPLC	High Performance Liquid Chromatography
H&E	Haematoxylin and Eosin
LP	Lysylpyridinoline
PBS	Phosphate-Buffered Saline
PBST	Phosphate-Buffered Saline-Tween
POP	Pelvic Organ Prolapse
RNA	Ribonucleic Acid
RT-PCR	Real Time-Polymerase Chain Reaction
SD	Standard Deviation
TCP	Tissue Culture Plate
