# Supplementary material for: Extracellular Matrix Stiffness and Composition Regulate the Myofibroblast Differentiation of Vaginal Fibroblasts
Source: Int J Mol Sci. 2020 Jul 4;21(13):4762. doi: 10.3390/ijms21134762 (PMC7369731; doi:10.3390/ijms21134762)
Supplement: Supplementary file 1 [file ijms-21-04762-s001.pdf]

Article

Extracellular Matrix Stiffness and Composition Regulate the Myofibroblast Differentiation of Vaginal Fibroblasts

Supplementary Materials

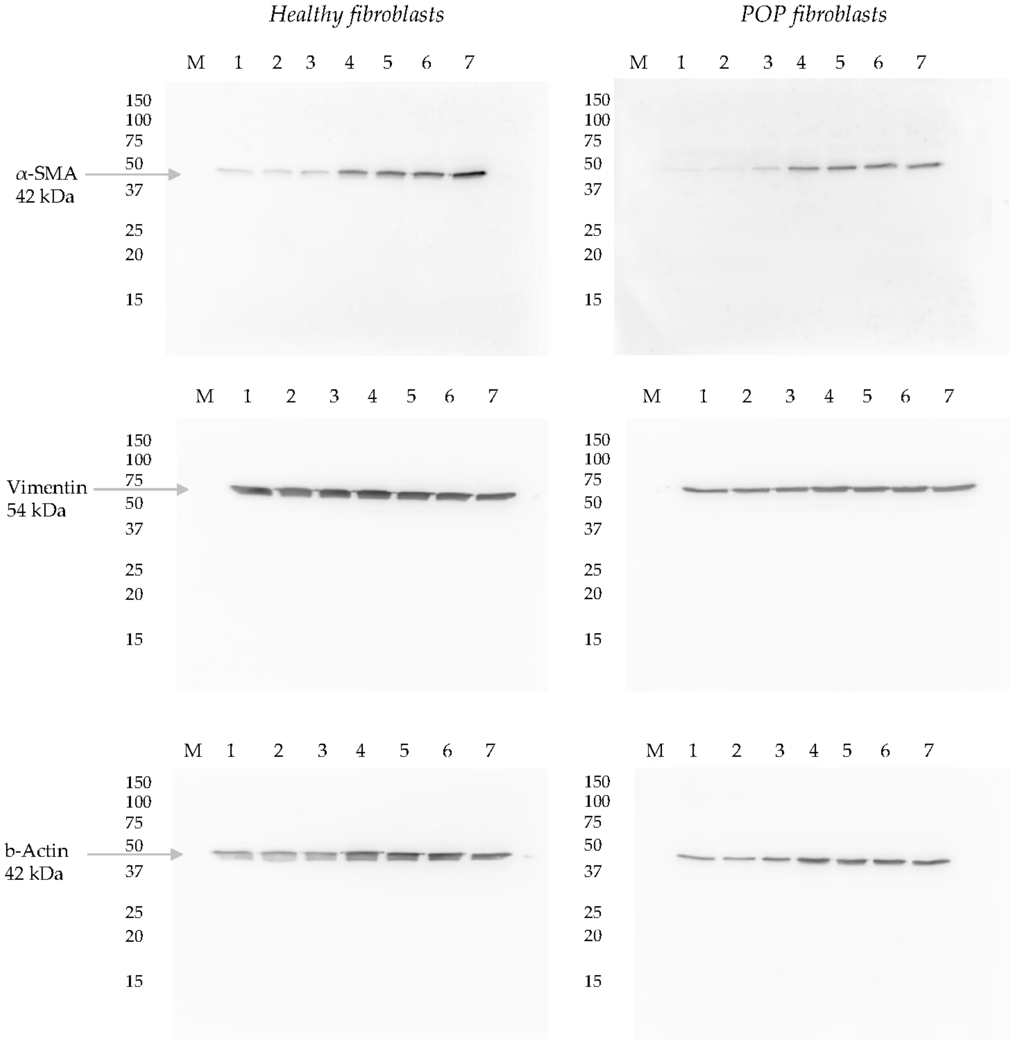

Codes used to identify the lanes:

M = Marker
1 = 0,2 kPa
2 = 0,5 kPa
3 = 2 kPa
4 = 8 kPa
5 = 16 kPa
6 = 32 kPa
7 = 64 kPa

Abreviations: α-SMA: aplha-smooth muscle actin; kPa: kilo Pascals; POP: pelvic organ prolapse

Figure S1. Original Western Blots images corresponding to Figure 1.

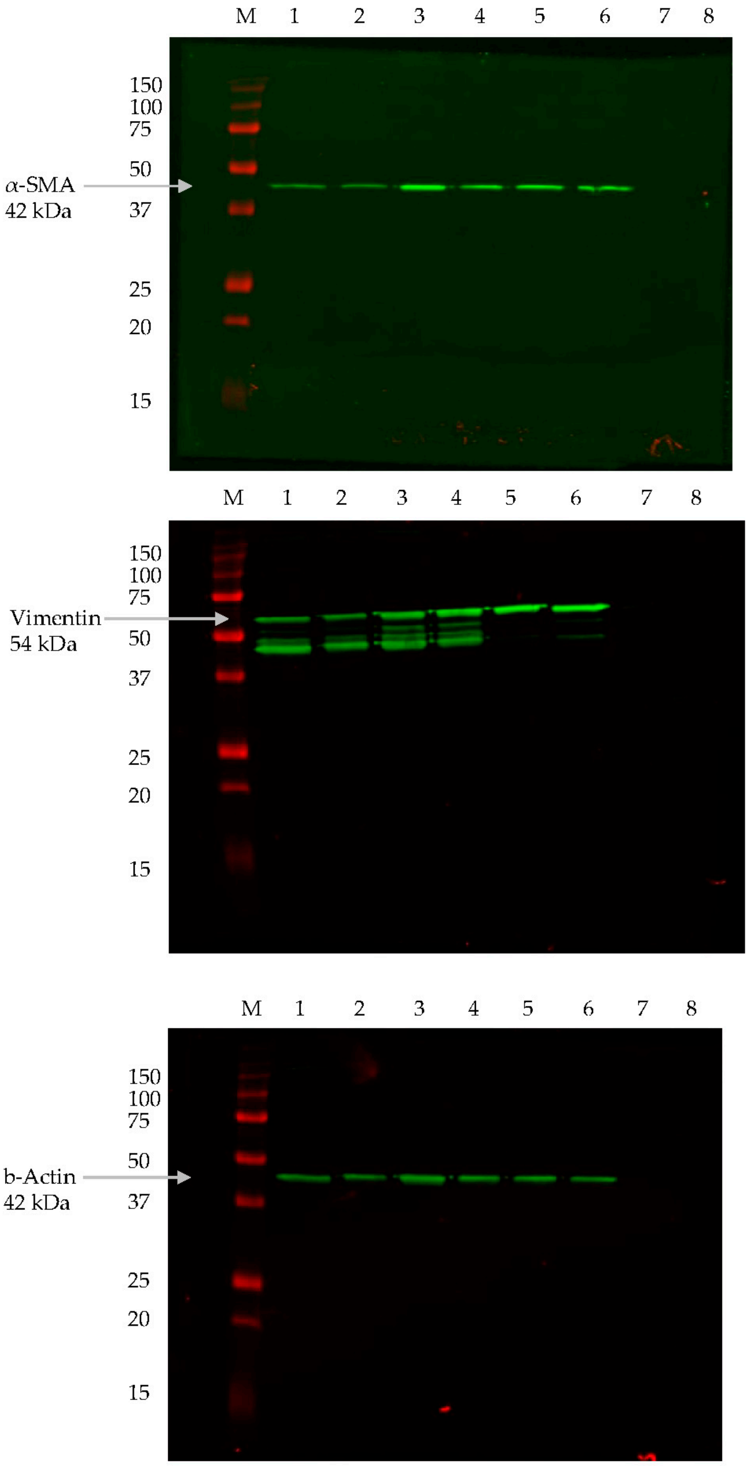

Codes used to identify the lanes:

M = Marker

1 = ECM-Healthy + Healthy fibroblasts

2 = ECM-Healthy + POP fibroblasts

3 = ECM-POP + Healthy fibroblasts

4 = ECM-POP + POP fibroblasts

5 = Healthy fibroblasts

6 = POP fibroblasts

7 = ECM-Healthy

8 = ECM-POP

Abreviations: α-SMA: aplha-smooth muscle actin; ECM: extracellular matrix; POP: pelvic organ prolapse

Figure S2. Original Western Blots images corresponding to Figure 4.
